# Supplementary material for: Neuronal DNA repair mediates systemic mitochondrial maintenance through acetylcholine signaling
Source: Nucleic Acids Res. 2026 Oct 26;54(20):gkag976. doi: 10.1093/nar/gkag976 (PMC13697224; doi:10.1093/nar/gkag976)
Supplement: Supplementary file 1 [file gkag976_supplemental_files.zip › Supplementary files.pdf]

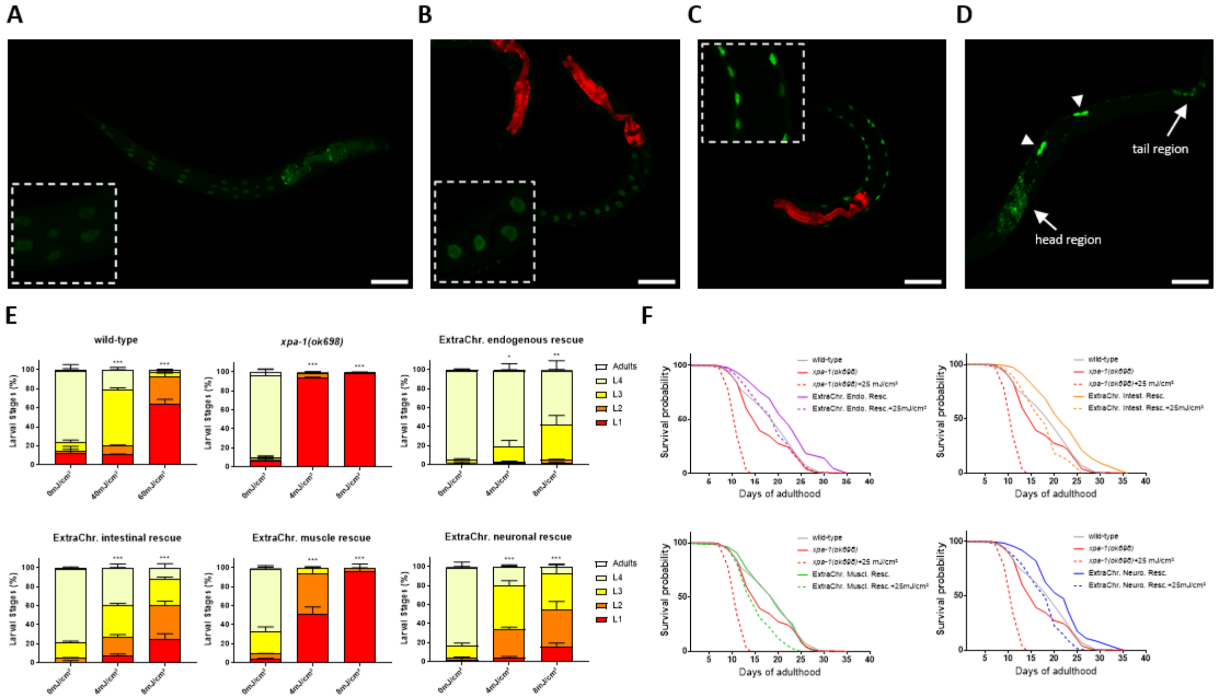

Supplementary Figure 1. L1 representation and extrachromosomal XPA-1::GFP expression rescues. (A) Representative fluorescence images of L1 nematodes showing XPA-1::GFP expression under the endogenous xpa-1 promoter. Scale bars = 10µm. (B) Representative fluorescence images of XPA-1::GFP expression in the nuclei of intestinal cells (pges-1::xpa-1::gfp) and tdTomato expression in the pharynx (pmyo-2::tdTomato) (co-injection marker), from L1 nematodes. Scale bar = 10µm. (C) Representative fluorescence images of XPA-1::GFP expression in the nuclei of muscle cells (pmyo-3::xpa-1::gfp) and tdTomato expression in the pharynx (pmyo-2::tdTomato) (co-injection marker)), from L1 nematodes . Scale bar = 10µm. (D) Representative fluorescence images of XPA-1::GFP expression in the nuclei of neuronal cells (prgef-1::xpa-1::gfp), And GFP expression in coelomocytes (punc-122::gfp) (co-injection marker) (white arrowheads), from L1 nematodes. Scale bar = 10µm.(E) Quantification of development of wild-type, xpa-1(ok698) and extrachromosomal re-expression of XPA-1 strains, 72 hours after UVB irradiation at L1 stage. Data represent Mean+SD of N=3 biological replicates. Statistical analysis: ANOVA. p-value:*<0.05, **<0.01, ***<0.001. (F) Lifespan assays of extrachromosomal re-expression of XPA-1 strains after UVB irradiation at day 1 adult stage. Representative survival curve from one individual experiment. n>100 per condition.

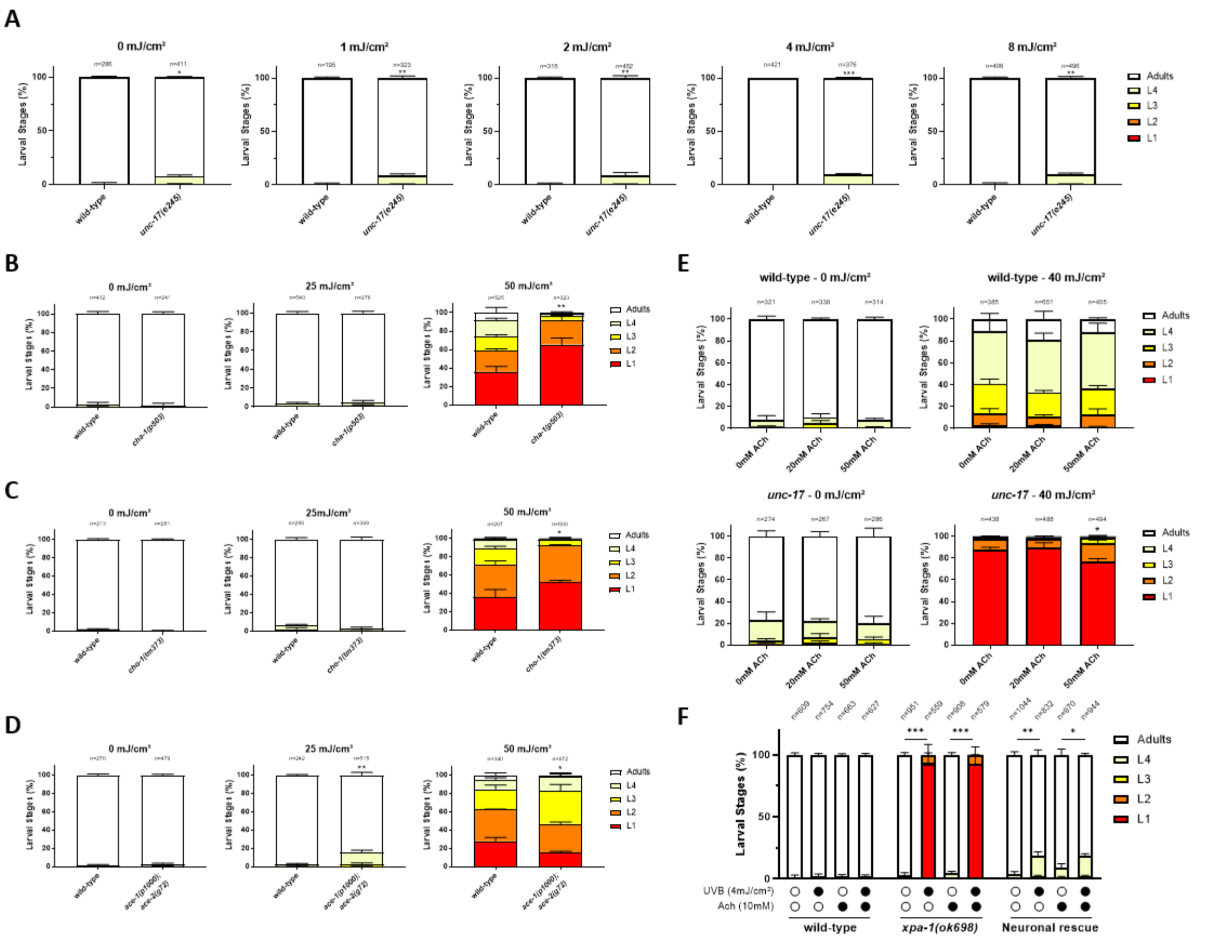

Supplementary Figure 2. Ach signaling impairment leads to DNA damage sensitivity but is not rescued by Ach supplementation. (A) Quantification of development of wild-type and acetylcholine signaling mutant *unc-17(e245)* strains 72 hours after UVB irradiation at L1 stage. Data represent Mean+SD of N=3 biological replicates. Statistical analysis: ANOVA. *p*-value: ***<0.001. **(B)** Quantification of development of wild-type and acetylcholine signaling mutant *cha-1(p503)* strains, 72 hours after UVB irradiation at L1 stage. Data represent Mean+SD of N=3 biological replicates. Statistical analysis: ANOVA. *p*-value:*<0.05, **<0.01, ***<0.001. **(C)** Quantification of development of wild-type and acetylcholine signaling mutant *cho-1(tm373)* strains, 72 hours after UVB irradiation at L1 stage. Data represent Mean+SD of N=3 biological replicates. Statistical analysis: ANOVA. *p*-value:*<0.05, **<0.01, ***<0.001. **(D)** Quantification of development of wild-type and acetylcholine signaling mutant *ace-1(p1000);ace-2(g72)* strains, 72 hours after UVB irradiation at L1 stage. Data represent Mean+SD of N=3 biological replicates. Statistical analysis: ANOVA. *p*-value:*<0.05, **<0.01, ***<0.001. **(E)** Quantification of development of wild-type and acetylcholine signaling mutant *unc-17(e245)* strains treated with 0, 20 or 50mM acetylcholine, 72 hours after UVB irradiation at L1 stage. Data represent Mean+SD of N=3 biological replicates. Statistical analysis: ANOVA. *p*-value:*<0.05, **<0.01, ***<0.001.

(F) Quantification of development of wild-type, NER deficient *xpa-1(ok698)* and Neuronal rescue strains treated with 10mM acetylcholine, 72 hours after UVB irradiation at L1 stage. Data represent Mean+SD of N=3 biological replicates. Statistical analysis: ANOVA. *p*-value:*<0.05, **<0.01, ***<0.001.

Strain	Genotype
N2	Bristol; wildtype
RB864	*xpa-1(ok698) I.*
BJS738	*xpa-1(ok698) I.; In[pges-1::gfp::xpa-1;pmyo-2::tdTomato]*
BJS740	*xpa-1(ok698) I.; In[pmyo-3::gfp::xpa-1;pmyo-2::tdTomato]*
BJS903	*xpa-1(ok698) I.; In[prgef-1::gfp::xpa-1;punc-122::gfp]*
BJS741	*xpa-1(ok698) I.; In[pxpa-1::gfp::xpa-1;pmyo-2::tdTomato]*
EG9631	*unc-13(s69) I.*
CB928	*unc-31(e928) IV.*
MT15620	*cat-2(n4547) II.*
MT6308	*eat-4(ky5) III.*
MT9455	*tbh-1(n3247) X.*
MT13113	*tdc-1(n3419) II.*
MT14984	*tph-1(n4622) II.*
CB933	*unc-17(e245) IV.*
CB156	*unc-25(e156) III.*
BJS902	*xpa-1(ok698) I.; cat-2(n4547) II.*
BJS922	*xpa-1(ok698) I.; eat-4(ky5) III.*
BJS912	*xpa-1(ok698) I.; tbh-1(n3247) X.*
BJS923	*xpa-1(ok698) I.; tdc-1(n3419) II.*
BJS914	*xpa-1(ok698) I.; tph-1(n4622) II.*
BJS918	*xpa-1(ok698) I.; unc-17(e245) IV.*
BJS919	*xpa-1(ok698) I.; unc-25(e156) III.*
BJS943	*xpa-1(ok698) I.; unc-17(e245) IV.; In[prgef-1::gfp::xpa-1;punc-122::gfp]*
PR1162	*cha-1(p503) IV.*
RM2576	*cho-1(tm373) IV.*
GG201	*ace-2(g72) I.; ace-1(p1000) X.*
CU5991	*fzo-1(tm1133) II.*
BJS1202	*xpa-1(ok698) I.; fzo-1(tm1133) II.; In[prgef-1::gfp::xpa-1;punc-122::gfp]*

Supplementary Table 1. Used and generated *C. elegans* strains.

Figure 2				
Panel	Sub panel	conditions	vs	p-value
B	wild-type	4mJ/cm²	0mJ/cm²	0,835093076
B	wild-type	8mJ/cm²	0mJ/cm²	0,562712343
B	wild-type	16mJ/cm²	0mJ/cm²	0,226501644
B	*xpa-1(ok698)*	4mJ/cm²	0mJ/cm²	2,25028E-05
B	*xpa-1(ok698)*	8mJ/cm²	0mJ/cm²	1,20511E-08
B	*xpa-1(ok698)*	16mJ/cm²	0mJ/cm²	1,20511E-08
B	Endogenous Rescue	4mJ/cm²	0mJ/cm²	0,32627159
B	Endogenous Rescue	8mJ/cm²	0mJ/cm²	0,001028448
B	Endogenous Rescue	16mJ/cm²	0mJ/cm²	0,006194415
B	Intestinal Rescue	4mJ/cm²	0mJ/cm²	0,000285774
B	Intestinal Rescue	8mJ/cm²	0mJ/cm²	6,8839E-06
B	Intestinal Rescue	16mJ/cm²	0mJ/cm²	2,51381E-05
B	Muscle Rescue	4mJ/cm²	0mJ/cm²	0,000967277
B	Muscle Rescue	8mJ/cm²	0mJ/cm²	0,002399331
B	Muscle Rescue	16mJ/cm²	0mJ/cm²	1,03178E-08
B	Neuronal rescue	4mJ/cm²	0mJ/cm²	0,053586099
B	Neuronal rescue	8mJ/cm²	0mJ/cm²	0,003430557
B	Neuronal rescue	16mJ/cm²	0mJ/cm²	1,40406E-08

Figure 4				
Panel	Sub panel	conditions	vs	p-value
B	0mJ/cm²	*unc-13(S69)*	wild-type	0,000112281
B	0mJ/cm²	*unc-31(e928)*	wild-type	0,323766414
B	25mJ/cm²	*unc-13(S69)*	wild-type	8,51407E-05
B	25mJ/cm²	*unc-31(e928)*	wild-type	0,256866035
B	50mJ/cm²	*unc-13(S69)*	wild-type	0,000650864
B	50mJ/cm²	*unc-31(e928)*	wild-type	0,107231026
C	0mJ/cm²	*cat-2(n4547)*	wild-type	0,323762402
C	0mJ/cm²	*eat-4(ky5)*	wild-type	0,042934953
C	0mJ/cm²	*tbh-1(n3247)*	wild-type	0,009832303
C	0mJ/cm²	*tdc-1(n3419)*	wild-type	0,012940532
C	0mJ/cm²	*tph-1(n4622)*	wild-type	0,001946718
C	0mJ/cm²	*unc-17(e245)*	wild-type	0,158852723
C	0mJ/cm²	*unc-25(e156)*	wild-type	0,003580866
C	25mJ/cm²	*cat-2(n4547)*	wild-type	0,077409911
C	25mJ/cm²	*eat-4(ky5)*	wild-type	0,013465246
C	25mJ/cm²	*tbh-1(n3247)*	wild-type	0,009236807
C	25mJ/cm²	*tdc-1(n3419)*	wild-type	0,001832779
C	25mJ/cm²	*tph-1(n4622)*	wild-type	0,001175434
C	25mJ/cm²	*unc-17(e245)*	wild-type	0,000171115
C	25mJ/cm²	*unc-25(e156)*	wild-type	0,000887449
C	50mJ/cm²	*cat-2(n4547)*	wild-type	0,117592551
C	50mJ/cm²	*eat-4(ky5)*	wild-type	0,173245163
C	50mJ/cm²	*tbh-1(n3247)*	wild-type	0,275600632
C	50mJ/cm²	*tdc-1(n3419)*	wild-type	0,319120574
C	50mJ/cm²	*tph-1(n4622)*	wild-type	0,012944621
C	50mJ/cm²	*unc-17(e245)*	wild-type	7,97465E-06
C	50mJ/cm²	*unc-25(e156)*	wild-type	0,124632008
D	0mJ/cm²	*cat-2;xpa-1*	*xpa-1*	0,608197117
D	0mJ/cm²	*eat-4;xpa-1*	*xpa-1*	0,006852127
D	0mJ/cm²	*tbh-1;xpa-1*	*xpa-1*	0,108371593
D	0mJ/cm²	*tdc-1;xpa-1*	*xpa-1*	0,169993671
D	0mJ/cm²	*tph-1;xpa-1*	*xpa-1*	0,023489128
D	0mJ/cm²	*unc-17;xpa-1*	*xpa-1*	0,000858369
D	0mJ/cm²	*unc-25;xpa-1*	*xpa-1*	0,155458693
D	1mJ/cm²	*cat-2;xpa-1*	*xpa-1*	0,316941717
D	1mJ/cm²	*eat-4;xpa-1*	*xpa-1*	0,306968478
D	1mJ/cm²	*tbh-1;xpa-1*	*xpa-1*	0,135729789
D	1mJ/cm²	*tdc-1;xpa-1*	*xpa-1*	0,084991219
D	1mJ/cm²	*tph-1;xpa-1*	*xpa-1*	0,179272344
D	1mJ/cm²	*unc-17;xpa-1*	*xpa-1*	0,000372265
D	1mJ/cm²	*unc-25;xpa-1*	*xpa-1*	0,189877722
D	2mJ/cm²	*cat-2;xpa-1*	*xpa-1*	0,569285777
D	2mJ/cm²	*eat-4;xpa-1*	*xpa-1*	0,023118746
D	2mJ/cm²	*tbh-1;xpa-1*	*xpa-1*	0,527948588
D	2mJ/cm²	*tdc-1;xpa-1*	*xpa-1*	0,236327276
D	2mJ/cm²	*tph-1;xpa-1*	*xpa-1*	0,613041237
D	2mJ/cm²	*unc-17;xpa-1*	*xpa-1*	0,005952114
D	2mJ/cm²	*unc-25;xpa-1*	*xpa-1*	0,160692612
H	0mJ/cm²	Neuronal Rescue;*unc-17(e245)*	Neuronal Rescue	0,014651715
H	4mJ/cm²	Neuronal Rescue;*unc-17(e245)*	Neuronal Rescue	5,0702E-05
H	8mJ/cm²	Neuronal Rescue;*unc-17(e245)*	Neuronal Rescue	0,006268349

Figure 5				
Panel	Sub panel	conditions	vs	p-value
G	wild-type	15mJ/cm²	0mJ/cm²	0,063814779
G	wild-type	30mJ/cm²	0mJ/cm²	1,50932E-05
G	*fzo-1(tm1133)*	15mJ/cm²	0mJ/cm²	1,94082E-05
G	*fzo-1(tm1133)*	30mJ/cm²	0mJ/cm²	1,94082E-05
H	0mJ/cm²	Neuronal Rescue	*xpa-1(ok698)*	0,286859881
H	0mJ/cm²	Neuronal Rescue;*fzo-1(tm1133)*	*xpa-1(ok698)*	0,45702447
H	8mJ/cm²	Neuronal Rescue	*xpa-1(ok698)*	0,000318114
H	8mJ/cm²	Neuronal Rescue;*fzo-1(tm1133)*	*xpa-1(ok698)*	0,695574675

Supplementary figure 1				
Panel	Sub panel	conditions	vs	p-value
E	wild-type	40mJ/cm²	0mJ/cm²	0,000351196
E	wild-type	60mJ/cm²	0mJ/cm²	0,000181602
E	*xpa-1(ok698)*	4mJ/cm²	0mJ/cm²	1,30475E-05
E	*xpa-1(ok698)*	8mJ/cm²	0mJ/cm²	1,10306E-05
E	Endogenous Rescue	4mJ/cm²	0mJ/cm²	0,049402637
E	Endogenous Rescue	8mJ/cm²	0mJ/cm²	0,004061036
E	Intestinal Rescue	4mJ/cm²	0mJ/cm²	0,000214634
E	Intestinal Rescue	8mJ/cm²	0mJ/cm²	0,000117831
E	Muscle Rescue	4mJ/cm²	0mJ/cm²	0,000147406
E	Muscle Rescue	8mJ/cm²	0mJ/cm²	1,59711E-05
E	Neuronal rescue	4mJ/cm²	0mJ/cm²	0,000165324
E	Neuronal rescue	8mJ/cm²	0mJ/cm²	0,000608929

Supplementary figure 2				
Panel	Sub panel	conditions	vs	p-value
A	0mJ/cm²	unc-17(e245)	wild-type	0,021449079
A	1mJ/cm²	unc-17(e245)	wild-type	0,004855657
A	2mJ/cm²	unc-17(e245)	wild-type	0,006062898
A	4mJ/cm²	unc-17(e245)	wild-type	1,47294E-05
A	8mJ/cm²	unc-17(e245)	wild-type	0,001439436
B	0mJ/cm²	*cha-1(p503)*	wild-type	0,669354402
B	25mJ/cm²	*cha-1(p503)*	wild-type	0,567482502
B	50mJ/cm²	*cha-1(p503)*	wild-type	0,009931459
C	0mJ/cm²	*cho-1(tm373)*	wild-type	0,065262439
C	25mJ/cm²	*cho-1(tm373)*	wild-type	0,158414982
C	50mJ/cm²	*cho-1(tm373)*	wild-type	0,01897918
D	0mJ/cm²	*ace-1(p1000);ace-2(g72)*	wild-type	0,147747646
D	25mJ/cm²	*ace-1(p1000);ace-2(g72)*	wild-type	0,002723306
D	50mJ/cm²	*ace-1(p1000);ace-2(g72)*	wild-type	0,031770178
E	wild-type - 0 mJ/cm²	20mM Ach	0mM Ach	0,360353755
E	wild-type - 0 mJ/cm²	50mM Ach	0mM Ach	0,718155185
E	wild-type - 40 mJ/cm²	20mM Ach	0mM Ach	0,342218437
E	wild-type - 40 mJ/cm²	50mM Ach	0mM Ach	0,623420456
E	unc-17 - 0 mJ/cm²	20mM Ach	0mM Ach	0,496623998
E	unc-17 - 0 mJ/cm²	50mM Ach	0mM Ach	0,490103789
E	unc-17 - 40 mJ/cm²	20mM Ach	0mM Ach	0,389759653
E	unc-17 - 40 mJ/cm²	50mM Ach	0mM Ach	0,015233484
F	wild-type	+UVB/-Ach	-UVB/-Ach	0,474679544
F	wild-type	+UVB/+Ach	-UVB/+Ach	0,808045582
F	*xpa-1(ok698)*	+UVB/-Ach	-UVB/-Ach	4,14908E-05
F	*xpa-1(ok698)*	+UVB/+Ach	-UVB/+Ach	1,74237E-05
F	Neuronal rescue	+UVB/-Ach	-UVB/-Ach	0,005706458
F	Neuronal rescue	+UVB/+Ach	-UVB/+Ach	0,021449079

Supplementary Table 2. Exact p-values of statistical analysis of developmental assays.
